# Efficacy of neem chippings for mosquito larval control under field conditions

**DOI:** 10.1186/s12898-015-0041-0

**Published:** 2015-03-08

**Authors:** Susan S Imbahale, Wolfgang R Mukabana

**Affiliations:** International Centre of Insect Physiology and Ecology, P.O. Box 30772 GPO, Nairobi, Kenya; Department of Applied and Technical Biology, Technical University of Kenya, P.O Box 52428, City Square, Nairobi, Kenya; School of Biological Sciences, University of Nairobi, P.O. Box 30197 GPO, Nairobi, Kenya

**Keywords:** Neem chippings, *Azadirachta indica*, *Bacillus thuringiensis israelensis*, *Bti*, Anopheline, Nyabondo, Botanicals, Culicine, Mosquitoes

## Abstract

**Background:**

An in depth understanding of mosquito breeding biology and factors regulating population sizes is fundamental for vector population control. This paper presents results from a survey of mosquito breeding habitats and the efficacy of neem chippings as a potential larvicide that can be integrated in mosquito control on Nyabondo Plateau in western Kenya.

**Results:**

Six main mosquito habitat types namely artificial ponds, abandoned fish ponds, active fish ponds, open drains, temporary pools and swamps were found in Nyabondo. Early anopheline instars were mainly recovered from temporary pools, artificial ponds and abandoned fish ponds. The mosquitoes collected were *Anopheles gambiae* sensu lato (35%), *An. coustani* (46%) and *Culex spp* (19%). Both early and late instar larvae of anopheline and culicine mosquitoes were more abundant in the controls than in the *Bti* and neem treated habitats. Within treated habitats, early instar anopheline mosquitoes were recovered more from habitats provided with neem and fish compared to *Bti* treated habitats. All treated habitats recorded higher numbers of early instar larvae than late instars or pupae, indicating that gravid female mosquitoes still oviposited within treated habitats.

**Conclusions:**

Neem chippings are a good tool for mosquito larval source management under field conditions. However, more research needs to be done to quantify the contribution of this tool to the overall mosquito borne disease transmission.

## Background

Mosquitoes are among the most devastating disease vectors in sub-Saharan Africa [1]. Their proliferation is mainly facilitated by climatic and ecological changes associated with developmental activities such us construction, farming, brick making etc. [2-8]. Mosquito vector control programmes have relied heavily on the use of insecticides through indoor residual spraying (IRS) and insecticide treated bed nets (ITNs) [1,9]. Both strategies focus on indoor adult mosquito vector populations because shortening the life of this developmental stage can have a major impact on vectorial capacity [10]. Furthermore, adult female mosquitoes make themselves vulnerable to control interventions when they seek for blood meals from human hosts [11]. Despite the successes reported in using insecticides for mosquito control, this is threatened by the rise of insecticide resistance in mosquito vectors [12], which necessitates the development and trialing of complementary and/or supplementary strategies. Although unlikely to replace insecticide-based adult mosquito control, larval control methods offer sustainable supplements to existing mosquito vector control efforts [13-16]. One advantage of targeting larvae is that they cannot escape from their breeding sites as an avoidance mechanism against control measures [14].

Mosquito larval control commonly referred to as larval source management (LSM) is particularly valuable in regions where the primary mosquito vectors are exophilic and/or bite before people are in bed, so rendering IRS and ITNs less effective [17-20]. Larval source management is the management of aquatic habitats that are potential larval habitats for mosquitoes, in order to prevent the completion of development of the immature stages [21]. Some LSM strategies that are widely used include (a) application of the spore-forming bacteria *Bacillus thuringiensis* var. *israelensis (Bti)* and *B. sphaericus* (*Bs*) that are very selective agents against mosquito and midge larvae [14,16,22-24], (b) environmental management [25] and (c) use of botanicals [26]. Although insecticides of plant origin have existed for many years, they have not been fully utilized against vectors of public health importance [27]. One of the most studied botanical sources is the neem tree *Azadiracta indica* whose extracts have shown considerable lethal activity against insect pests of agricultural and public health importance [28-31]. The LSM strategies available for the control of mosquito larvae can only be effective when tailored appropriately to local ecology and infrastructure [32]. This study was carried out on Nyabondo Plateau, a rural setting in western Kenya, to (a) characterize mosquito breeding habitats and (b) assess the efficacy of neem chippings as a mosquito larval source management strategy.

## Methods

### Study area

This study was carried out on Nyabondo Plateau. Nyabondo plateau is located in Upper Nyakach Division, Nyando sub county, western Kenya. The plateau lies at an altitude of 1,658 m above sea level and at 0° 23′ 0 S and 34° 58′ 60 E. Upper Nyakach Division has a population density of 368 persons per square km and an estimated population size of 332,313 persons [33]. The population is dominated by the local *Dholuo* speaking people. The local inhabitants depend on brick making and selling as the main economic activity. Small scale crop and animal husbandry are also practiced. Crop agriculture is dominated by maize, bananas, cassava, sorghum and sweet potatoes. Domesticated animals mainly include cattle, sheep, goats and donkeys. Previous entomological surveys in Nyabondo found that larval *Anopheles* mosquitoes bred in both temporary and permanent habitats. Adult *An. gambiae* complex mosquitoes collected during the surveys found *An. arabiensis* (99.3%) to be the main malaria vector species, followed by *An. gambiae* (0.7%) [34].

### Mosquito larval sampling

The primary entomological outcome of this study was the presence of immature mosquitoes which served to evaluate the effectiveness of various larval control treatments. Larval sampling was done once weekly using a sweep net [6]. Up to a maximum of five sweeps were taken along the edges of each habitat depending on its surface area and size. Sweeps were taken until no more organisms, visible to the naked eye, were being observed. Sweep net contents were emptied into a white tray to enhance visibility, identification and counting of sampled organisms. About 10 minutes was allowed for the water to settle down before the next sweep was made. Collected specimens were sorted into mosquito larvae and other aquatic organisms. Mosquito larvae were sorted into anopheline and culicine subtypes, counted and recorded as early instars (L1 and L2), late instars (L3 and L4) or pupae. Pupae were not separated into the anopheline and culicine subtypes. Other organisms were returned back into the water after recording their identity and numbers. Collected pupae were transported to the field laboratory of the International Centre of Insect Physiology and Ecology (*icipe’*s) located at the Nyabondo Mission Hospital where they were allowed to emerge into adults before being identified taxonomically [35].

### Mosquito larval habitat characterization

The aim of these investigations was to assess the diversity of mosquito larval breeding habitats on Nyabondo plateau so as to inform any current and future larval source management endeavors. All potential breeding habitats were identified and checked for the presence or absence of mosquito larvae. In addition habitat type, size and origin (i.e. if man made or natural) plus water movements and presence or absence of aquatic vegetation within the habitats was determined and recorded.

### Efficacy of neem chippings as a mosquito larval control agent

The main aim of these investigations was to assess the efficacy of neem chippings as a mosquito larval control agent under field conditions. The work was carried out on Nyabondo plateau in western Kenya. Four treatments were used in these evaluations. The treatments included neem chippings, mosquito predatory fish (*Oreochromis niloticus*), the bio-larvicide *Bacillus thuringiensis israelensis* (*Bti*), and untreated (negative) controls. The negative controls included an assortment of mosquito larval habitats none of which was treated with neem chippings, mosquito predatory fish or *Bti*. Habitat types in the negative control category included abandoned fish ponds, artificial ponds, open drains, swamps and temporary pools such as brick pits.

The test treatment i.e. neem chippings were prepared from the stem of the neem tree *Azadirachta indica* [36], which belongs to the Family Meliaceae. The crude neem chippings were packed in 30 × 50 cm nylon bags each weighing about 1.5 kg. Only one such bag was placed in each artificial pond habitat. A total of 10 artificial ponds were treated with neem bags. The pesticidal activity of neem is attributed to Azadirachtin, which is the active eco-friendly ingredient [37-39]. Most efficacy studies have concentrated on using neem seed and leaf extracts in which Azadirachtin is most concentrated [28]. One school of thought considers the crude neem plant as being less expensive and effective than the purified compounds or extracts for controlling insects of medical and veterinary importance [40]. *Oreochromis niloticus* (Perciformes: Cichlidae), an edible fish species commonly known as Tilapia and which is farmed and eaten in the Nyabondo area, was used as a biological control agent. This acted as a *pseudo* positive control because the ten fish ponds that were selected and stocked with fingerlings of Tilapia (4 fish/m^2^) were not regularly cleared off of emergent vegetation as would be desired when using fish as a mosquito larval control agent [3]. Water-dispersible and granulated formulations of the commercial larvicide VectoBac® containing *Bacillus thuringiensis* var. *israelensis* (*Bti*; Valent Biosciences Corporation, Libertyville, IL, USA), i.e. the true positive control, was applied to all other temporary habitats and selected permanent habitats that contained mosquito larvae on Nyabondo plateau. The *Bti* was broadcasted on the larval habitats at weekly intervals at an optimum dosage and concentration of 200 g/ha as done elsewhere [37].

All experimental mosquito habitats were inspected once weekly for presence or absence of mosquito larvae.

### Ethical considerations

Ethical approval for this study was given by the Kenya National Ethical Review Committee located at the Kenya Medical Research Institute (SSC Protocol number 2675). A private land tenure system where individual families have exclusive rights to residential and agricultural parcels is observed in Nyabondo. Thus, verbal consent to carry out the research outlined in this article was sought from families.

### Data analysis

All raw data were entered in Microsoft Excel spreadsheets from where they were exported into the Statistical Package for Social Scientists (SPSS version 16.0) for analysis. The mosquito larval habitat characteristics recorded included presence or absence of mosquito larvae, the mosquito larval species type, presence or absence of water movements, presence or absence of aquatic vegetation, habitat type and origin. These data were explored using descriptive statistics functions in the SPSS. Chi-square analysis was used to determine the impact of variables collected on the presence or absence of immature mosquitoes within the different habitats. Binary regression was used to quantify the impact of significant variables on the presence of immature stages of mosquitoes. The numbers of mosquito larvae collected in the test treatment and the positive controls were statistically compared to those collected from the negative control treatments using posthoc Bonferroni tests.

## Results

The study was done from January to December 2012. Nyabondo plateau was searched and all stagnant waters checked for the presence or absence of mosquito larvae in January 2012. Experiments testing the efficacy of neem chippings as a mosquito larval control agent under field conditions were carried out from February to December 2012.

### Mosquito larval habitat characterization

**Figure 1:**
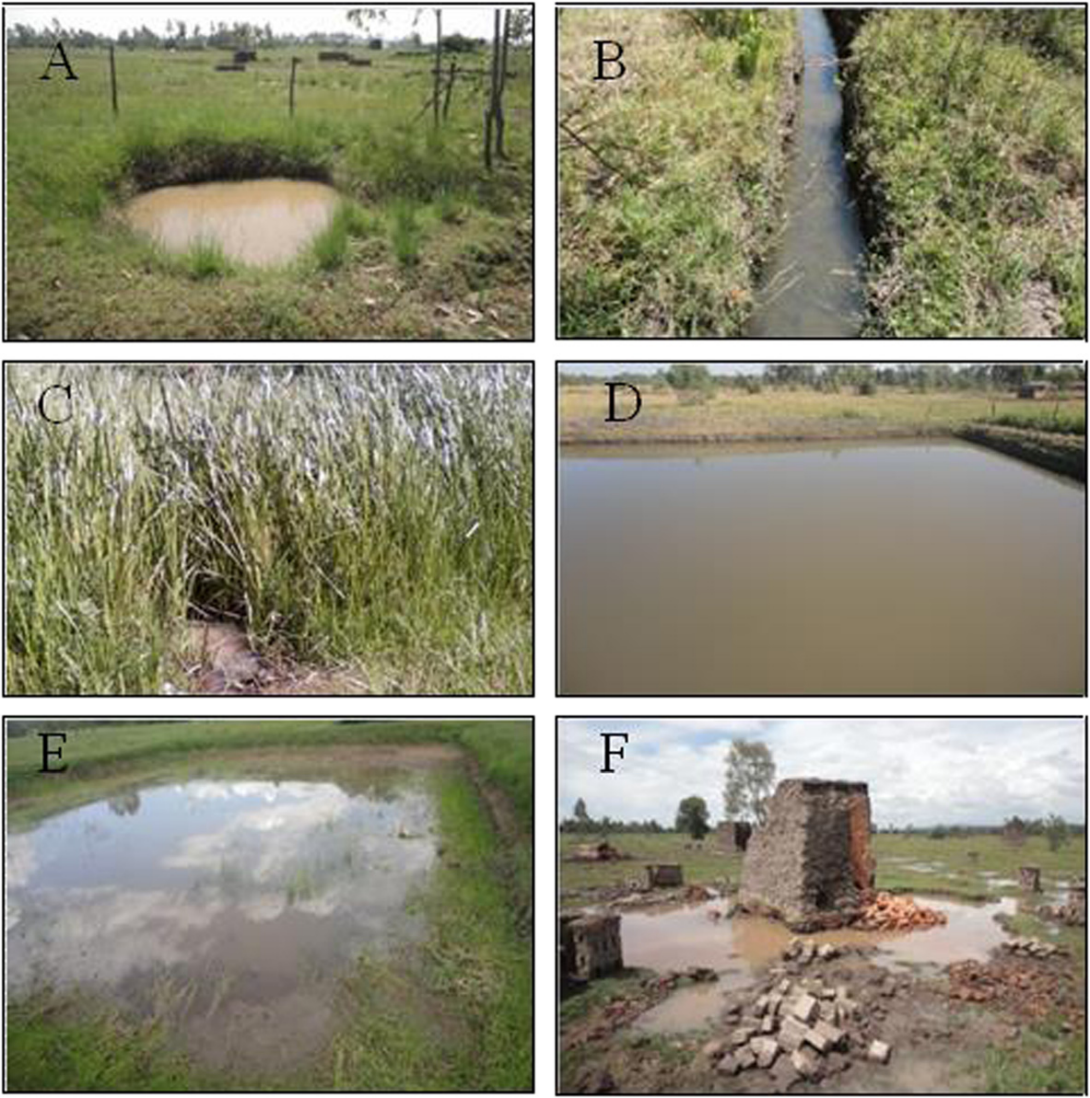
**Physical appearance of mosquito larval habitat types identified on Nyabondo plateau in western Kenya.** The habitats included artificial ponds **(A)**, open drains **(B)**, swamps **(C)**, active fish ponds **(D)**, abandoned fish ponds **(E)** and temporal pools **(F)**.

A total of 339 (N) potential habitats were identified among which 57% had mosquito larvae. The rest (43%) had no larvae. A total of six mosquito larval habitat types were identified (Figure 1). These included artificial ponds, open drains, swamps, active fish ponds, abandoned fish ponds and temporary pools (Table 1). Temporary pools were composed of foot/hoof prints, tire tracks, brick pits and ground pools. Temporary pools held water for approximately two weeks after rains and dried out when rains ceased. Permanent habitats held water for approximately 2–3 months after the rains. Majority of the habitats sampled were permanent (62%) in nature, with sizes ranging from 10 - 100 m in surface perimeter (72%) and occurring as a result of human activities (96%). Exploratory statistical analyses found the presence or absence of mosquito larvae inside habitats to be affected by habitat type (χ^2^ = 21.974; df = 5; P = 0.001), habitat stability i.e. whether the habitats were temporary or permanent (χ^2^ = 22.317; df = 1; P = 0.001), source of water (χ^2^ = 5.254; df = 1; P = 0.022), habitat size (χ^2^ = 9.822; df = 2; P = 0.007) and vegetation (χ^2^ = 12.547; df = 1; P = 0.001) (Table 2). Habitat origin i.e. whether natural or manmade (P = 0.350) and water movement within habitats (P = 0.381) had no effect on presence or absence of mosquito larvae (Table 2). Further analysis using binary logistic regression found habitat stability (temporary or permanent) to be the only significant determinant of the presence or absence of mosquito larvae in habitats (OR 0.353; C.I. 0.203 - 0.614; P = 0.001). Permanent habitats had a 65% higher chance of containing mosquito larvae than temporary habitats.

**Table 1:** **Mosquito larval habitat types found on Nyabondo plateau in western Kenya**

**Habitat Type**	**N (percent)**	**Description**
Artificial pond:	88 (70.5%)	A small manmade depression containing still water stored for local purposes e.g. brick making, watering cattle, domestic use etc.
Open drain:	50 (42.0%)	A channel dug to draw off water.
Swamp:	15 (66.6%)	A low-lying wet land with tall reeds and grassy vegetation
Active fish pond:	6 (0.0%)	A controlled inland body of standing freshwater stocked with fish
Abandoned fish pond:	68 (63.2%)	An unmaintained and/or uncontrolled inland body of standing freshwater with or without fish
Temporary pools:	112 (47.3%)	Shallow depressions holding water only during rains e.g.
		(a) *foot prints:* grooves made on the ground by man and/or cattle
		*(b) tire tracks:* grooves made on the ground by vehicles
		*(c) brick pits:* depressions made by digging out topsoil to make bricks
		*(d) ground pools*: naturally formed small area with still water

**Table 2:** **Factors affecting presence of mosquito larvae in breeding habitats on Nyabondo plateau, western Kenya**

**Factor**	**Factor level**	**Frequency (n)**	**Percentage**	**Chi-square**	**df**	**Significance**
Habitat stability	Permanent	214	63.13			
	Temporary	125	36.87	22.317	1	0.000
Habitat origin	Man-made	326	96.17			
	Natural	13	3.83	0.873	1	0.350
Water origin	Surface run-off	279	82.30			
	Underground	60	17.70	5.254	1	0.022
Habitat size	<10	45	13.27			
	>100	50	14.75			
	10 - 100	244	71.98	9.822	2	0.007
Water movement	No	301	88.79			
	Yes	38	11.21	0.768	1	0.381
Vegetation	Absent	149	43.95			
	Present	190	56.05	11.547	1	0.001

### Efficacy of neem chippings as a mosquito larval control agent in the field

**Figure 2:**
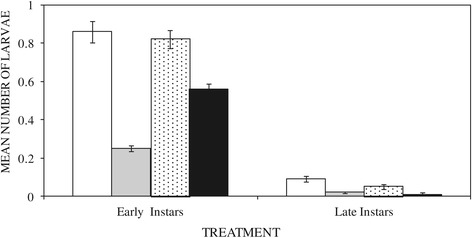
**Mean numbers of early and late anopheline larvae in untreated habitats (empty bar) or in habitats treated with the commercial biolarvicide** ***Bacillus thuringiensis israeliensis*** **(gray bar), the edible fish** ***Oreochromis niloticus*** **(hatched bar) and chippings of the neem tree** ***Azadiracta indica*** **(black bar).**

A total of 7,782 (93% early and 7% late instar) anopheline and 11,590 (71% early and 29% late instar) culicine mosquito larvae were sampled during the study. The presence or absence of larvae varied significantly among habitats assigned different treatments (P = 0.001). Early instar anopheline mosquitoes had 54.8%, 5.6% and 34% higher probabilities of being present in the negative controls compared to *Bti* (OR 0.452; C.I. 0.401 – 0.509; P = 0.001), fish (OR 0.944; CI 0.652 – 1.366; P = 0.759) and neem (OR 0.659; C.I. 0.531 – 0.820; P = 0.001) treated habitats, respectively (Figure 2). There were significantly higher numbers of early anopheline instars in habitats treated with neem (mean difference = 0.31 ± 0.061; P = 0.001) and fish (mean difference =0.57 ± 0.096; P = 0.001) than those treated with *Bti*. On the other hand the control had 77.4%, 59.8% and 84.5% higher chance of containing late anopheline larvae compared to *Bti* (OR 0.226 C.I. 0.169 – 0.302 P < 0.05), fish (OR 0.402 C.I. 0.188 – 0.860 P < 0.05) and neem (OR 0.155 C.I. 0.066 – 0.362 P < 0.05) treated habitats, respectively (Figure 2). There were no significant differences in late anopheline instars within habitats treated with neem, *Bti* and fish (P > 0.05).

**Figure 3:**
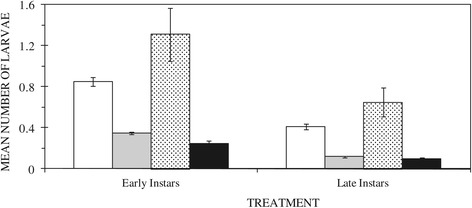
**Mean numbers of early and late culicine mosquito larvae in untreated habitats (empty bar) or in habitats treated with the commercial biolarvicide** ***Bacillus thuringiensis israeliensis*** **(gray bar), the edible fish** ***Oreochromis niloticus*** **(hatched bar) and chippings of the neem tree** ***Azadiracta indica*** **(black bar).**

Early culicine larvae were likely to be present in control rather than *Bti* (OR 0.466; C.I. 0.419 – 0.518; P = 0.001) and neem (OR 0.422; C.I. 0.342 – 0.521; P = 0.001) treated habitats (Figure 3). There were significantly higher numbers of early culicine instars in habitats treated with fish than those treated with *Bti* (mean difference = 0.96 ± 0.085; P = 0.001) and neem (mean difference = 1.06 ± 0.099; P = 0.001). No significant differences in numbers of early culicine instars were observed in habitats treated with neem and *Bti* (P > 0.874). Late culicine larvae were likely to be present in control rather than *Bti* (OR 0.305; C.I. 0.268 – 0.348; P = 0.001) and neem (OR 0.279; C.I. 0.210 – 0.370; P = 0.001) treated habitats (Figure 3). Similar to early instars, significantly higher numbers of late culicine instars were in habitats treated with fish than those treated with *Bti* (mean difference = 0.53 ± 0.053; P = 0.001) and neem (mean difference = 0.55 ± 0.062; P = 0.001). No significant differences in numbers of late culicine instars were observed between habitats treated with neem and *Bti* (P > 1.0).

**Figure 4:**
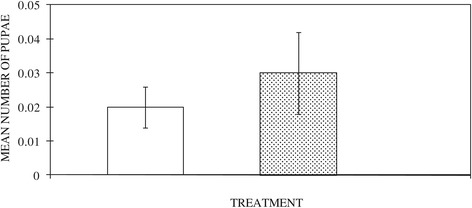
**Mean numbers of mosquito pupae in untreated habitats (empty bar) and in habitats treated with the edible fish** ***Oreochromis niloticus*** **(hatched bar).** No pupae were found in habitats treated with the commercial biolarvicide *Bacillus thuringiensis israeliensis* or with chippings of the neem tree *Azadiracta indica*.

A total of 145 pupae were collected with 70% success in adult emergence. Pupae were more likely to be present in control habitats than habitats exposed to treatments (Figure 4). Among the mosquitoes that emerged successfully were *An. gambiae* sensu lato (35%), *An. coustani* (46%) and culicine (19%) mosquito species. The presence of pupae was not different among the habitats except for *Bti*-treated habitats (OR 0.393; C.I. 0.229 – 0.674; P = 0.001), which had a 60.7% reduced chance of having pupae compared with the controls.

The other aquatic organisms that were encountered during field sampling included hemiptera, coleoptera and ephemeroptera among others.

## Discussion

Mosquito breeding habitats in Nyabondo varied from temporary pools, artificial ponds, open drains, swamps, abandoned and active fish ponds. The presence or absence of immature mosquitoes within the different habitat types varied depending on habitat stability i.e. whether permanent or temporary, source of water, habitat size and vegetation cover. More anopheline larvae were encountered in artificial ponds, temporary pools and abandoned fish ponds, whereas culicine larvae were likely to be present in artificial ponds, abandoned fish ponds and swamps. Among the mosquito species recorded in the area were *An. gambiae* sensu lato, *An. coustani* and *Culex* species. This is the first time *An. coustani* is being reported in Nyabondo. All treated and untreated habitats contained early instar larvae. However, habitats provided with treatments had reduced numbers of late instars of both anopheline and culicine mosquitoes. In general habitats treated with neem or *Bti* compared well showing no differences among all the stages and species.

Mosquito breeding habitats have been documented in many parts of Western Kenya [4-7,41]. Existing literature shows that breeding habitats are heterogeneous and that they vary from one location to another even in the same region. On Nyabondo Plateau the major breeding habitats were artificial ponds followed by temporary pools and abandoned fish ponds, all resulting from human activity. Artificial ponds were more abundant on the plateau as they are mostly used by brick makers for water preservation but unfortunately they provide the most preferred breeding habitats for mosquitoes. Apart from artificial ponds, early anopheline larvae were more often sampled from abandoned ponds, temporary pools and open drains. Culicine larvae outnumbered anophelines where they occurred in the same habitats except in the open drains. Habitats such as abandoned fish ponds and artificial ponds held water for long periods of time, which might have allowed for the establishment of a predator population that fed on the mosquito larvae before they developed into pupae. Field observations during sampling found such habitats to contain tadpoles, Hemiptera, Coleoptera and Ephemeroptera among the known mosquito predators and/or competitors. Secondly, the larvae were in most occasions more than the pupae, suggesting there may have been attrition resulting from natural competition for available resources. In this study it was assumed that the productive habitats, which contained more immature mosquitoes, produced more adult mosquitoes. Therefore, more larvae meant more biting population of mosquitoes and consequently an increased risk of mosquito borne disease. The assumption is supported by the findings by Ndenga and colleagues [6], who found the most productive habitats for adult mosquitoes to be those that recorded higher numbers of larvae.

All treated habitats recorded higher numbers of early instar larvae than late instars or pupae, indicating that gravid female mosquitoes still oviposited within treated habitats, exposing their progeny to lethal effects of treatments. Compared to the control, larvae from neem treated habitats had a much lower probability of growing to maturity because extremely few late instars and pupae of both species groups were present in these habitats. These findings support those of Howard and others [40], who reported that neem did not deter ovipositing females in a laboratory trial. Crude neem or partially-purified plant extracts which are less expensive, have been found to be highly effective for the control of mosquitoes rather than the purified compounds or pure extracts of the plant [40]. Crude extracts also discourage the development of resistance in the vectors [28]. The stem was not the only important part but the order of larvicidal potency among other parts of the plant was found to increase from the leaf to root to seed to bark [42]. This study is among the few that have reported field application of crude neem extracts. The application of neem chippings for the control of mosquitoes is encouraged because it minimizes the accumulation of harmful residues in the environment.

The bio-larvicide (*Bti*) was applied to all temporary habitats and a selected number of permanent habitats making up 71% of the proportion of habitats under this intervention. This was purposely done as *Bti* being the true positive control, has already been shown to work effectively. Consistent with existing literature on *Bti* [14-16,24,43], the immature anopheline and culicine mosquito populations were significantly reduced within the respective habitats. Habitats treated with *Oreochromis niloticus* had both early and late instars of anopheline and culicine mosquitoes throughout the sampling period. However, culicine larvae were more abundant in the presence of fish than in the control habitats suggesting that these could have been a less preferred mosquito species group compared to the anophelines. Predatory fish though suitable for use in artificial pond habitats was not as effective as the neem chippings and *Bti*. This may be because the active fish ponds used in this study were not regularly maintained to remove any emergent vegetation where larvae would hide. Although the field trials carried out demonstrate that neem chippings can contribute to a reduction in mosquito abundance, its impact on disease transmission remains a gap. Nevertheless, this study has clearly described mosquito breeding habitats and the efficacy of neem chippings as a mosquito larval control agent under field conditions.

## Conclusion

Neem chippings can be used as a locally available alternative that compliments well proven mosquito larval control agents namely the commercial bio-larvicide *Bacillus thuringiensis israelensis* (*Bti*). However, more research needs to be done to quantify the contribution of neem chippings to the overall mosquito borne disease transmission under field conditions.
